# Improving Cognitive Abilities in School-Age Children via Computerized Cognitive Training: Examining the Effect of Extended Training Duration

**DOI:** 10.3390/brainsci13121618

**Published:** 2023-11-22

**Authors:** Eugene H. Wong, Kevin P. Rosales, Lisa Looney

**Affiliations:** Department of Child Development, California State University, San Bernardino, CA 92407, USA; ewong@csusb.edu (E.H.W.); lisa.looney@csusb.edu (L.L.)

**Keywords:** computerized cognitive training, working memory, processing speed, cognitive flexibility, school setting

## Abstract

Critical neuropsychological capabilities such as working memory, cognitive flexibility, and processing speed are foundational to many daily activities. For children, such skills are essential for school success. Thus, children who demonstrate weaknesses in these abilities may experience impaired academic performance; this is especially true for students identified with learning differences who often exhibit less developed cognitive abilities. The purpose of this project was to examine the efficacy of a cognitive training program implemented during the school day to improve abilities predictive of academic achievement. Ninety-five children completed two training activities that were counterbalanced across participants. Analyses of baseline working memory, cognitive flexibility, and processing speed performance relative to those following training showed a strong treatment effect. Moreover, there is notable evidence of greater intervention efficacy with extended engagement with the training program. Implications for neuropsychological research and practice are discussed.

## 1. Introduction

An extensive body of the literature has been devoted to understanding working memory (WM), which is the ability to actively maintain and store information in the presence of complex cognition [1]. The reason for this is the ubiquitous presence that WM holds in theories of cognition [2] and in predicting real-world outcomes like reading comprehension [3], educational attainment [4], and intelligence [5]. For school-age children in the classroom, WM is often found to be a critical cognitive skill necessary for successful academic performance in many content areas like math, reading, and writing [6,7,8]. 

While WM is an important cognitive skill for a myriad of experiences, it is not the only cognitive ability that is important for academic performance. Cognitive flexibility (CF) and processing speed (PS) are also other notable aspects of cognition that have been examined in this vein of research, especially as it pertains to school-age children’s academic achievement. Both are also related to WM [9]. CF is defined as the skill necessary to effectively adapt thought to different mental sets or task rules [10]. For example, in a typical school day, children must transition from one subject area to the next (e.g., being able to process digits on a math worksheet while shortly after being required to produce a paragraph, which involves processing and producing verbal information such as words and sentences). Thus, possessing well-developed CF is important. On the other hand, PS is defined as how quickly an individual processes information [11]. Like WM and CF, children depend on PS in school-related scenarios where there are time demands. For example, school-age children often are tasked with completing math problems within a short timeframe. To be able to do so, PS becomes critical for children. Taken together, WM, CF, and PS are interrelated cognitive skills essential for school-age children in the academic environment. Much of the work highlighted above has focused on typically developing children among whom there would not be notable deficits in these cognitive abilities. However, it is important to examine deficits in WM, CF, and PS as such limitations likely impact school performance in a negative manner.

### 1.1. Working Memory, Cognitive Flexibility, and Processing Speed Deficits

For atypically developing children (who commonly are identified with learning differences), research has generally shown that academic performance is subpar relative to their typically developing counterparts. For example, Maehler and Schuchardt [12] investigated the degree to which children who possessed learning differences experienced impaired WM. In their study, they sampled children who had been diagnosed with dyslexia, dyscalculia, and attention-deficit hyperactivity disorder (ADHD). Among these children, measures of core components of WM (i.e., the phonological loop, visuospatial sketchpad, and central executive) showed deficits that were related to classroom challenges/struggles [12]. Furthermore, Gathercole et al. [13] assessed the relationship between WM and a national curriculum assessment in 4–7-year-old children. They reported that children with higher levels of WM performed well in math and English. The converse was also true, where children who displayed lower levels of WM performed less strongly on measures of math and English [13]. Similar findings are reported elsewhere [14,15,16].

Like WM, there is evidence that CF impacts academic performance. Filipetti and Krumm [17] examined the relations among CF, WM, shifting, creativity, and academic achievement (i.e., reading and writing). The authors reported that CF is related to both reading and writing. Specifically, individuals who displayed higher levels of CF performed better on reading and writing tasks while those who displayed lower levels of CF performed less well on the same achievement measures. The authors attributed the findings to the fact that reading and writing both require the ability to switch between different mental sets and rules. In the case of reading comprehension, students are continuously switching between content already read and content being read currently. As such, having efficient CF is necessary to effectively derive meaning from what is read. Writing also involves the ability to efficiently switch between different ideas to compose a paragraph or an essay; thus, CF is very important to the writing process. The authors provide evidence for the important role of CF in supporting academic achievement [17].

Deficits in PS appear to have similar effects on academic achievement. Using structural equation modeling (SEM), Dodonova and Dodonov [18] tested the structural relations between PS, intelligence, and academic achievement (math and language) in school-age children. Their results indicated that PS was moderately associated with school achievement (*r* = −0.43)—faster PS led to higher academic achievement and vice versa. These results indicate that about 18% of the variance in academic achievement was explained by PS [18] thus, underscoring a notable role of PS in predicting academic achievement. Other studies have reported similar findings. For example, Tikhomirova et al. [19] tested a model where PS, WM, number sense, and fluid intelligence predicted performance on school achievement tests. The results showed that PS indirectly impacted performance on measures of math and language achievement through WM, number sense, and fluid intelligence [19].

Taken together, there is evidence that deficits in WM, PS, and CF can impact academic achievement. In response to these findings, researchers have increasingly considered the efficacy of computerized cognitive training as an intervention that will remediate cognitive ability deficits.

### 1.2. The Malleability of Cognitive Skills and Computerized Cognitive Training

While it has been established that children (particularly those with learning differences; [12,13]) who display less-developed cognitive skills often suffer impaired performance in important academic subject areas like math, reading, and writing [16,17,18,19], efforts to remediate these deficits require a level of malleability in cognitive skills. That is unless these cognitive skills are plastic, efforts to improve children’s abilities will fall short. For decades, cognitive abilities were thought to be fixed; thus, the assumption was that academic performance negatively impacted by underdeveloped cognitive skills could not be improved. However, a growing body of empirical work has shown that skills like WM, for example, are malleable and can be improved via computerized cognitive training (CCT). In a notable study, Olesen et al. [20] sought to investigate whether engaging in cognitive training exercises led to neurological changes in brain structures associated with WM using functional magnetic response imaging (fMRI) technology. Participants engaged in cognitive exercises designed to tax WM for a period of five weeks. Both neurological and behavioral assessments of cognitive performance were administered before and after the cognitive training. Olesen and colleagues reported a number of important findings. First, there was increased activity in areas associated with WM functioning, including the frontal gyrus, right parietal cortex, and intraparietal cortex [20]. All these regions are housed in the prefrontal and parietal cortices. Second, the authors reported improvement in behavioral measures of WM at the posttest [20]. The findings documented by Olsen and colleagues were among the first to show evidence for neuroplasticity of WM.

To corroborate the above findings, Buschkuehl et al. [21] more recently tested the neural changes (both functional and resting) associated with short-term n-back training. The n-back task has traditionally been utilized to measure WM as it taxes important components of WM like updating and attention control [22]. Buschkuehl and colleagues had participants train 20 min per day for a span of 7 days. Measures of WM, specifically, visual and auditory n-back tasks were administered; additionally, brain images of neural functioning were collected. Results showed that for the behavioral outcomes, performance on the verbal and spatial n-back tasks improved at the posttest for the experimental group. No improvements were reported for the control group. Importantly, there was increased activation and perfusion of prefrontal brain regions [21]. In sum, studies showed that WM is malleable and can be improved via cognitive training. Not surprisingly, these projects served as a catalyst for other work that examined whether CCT can remediate WM deficits in children with learning differences.

### 1.3. Remediating WM Deficits in Children with Learning Differences

The established importance of certain cognitive skills (e.g., WM, CF, and PS) for successful academic performance coupled with the findings that some cognitive skills are malleable led to a line of research focusing on the use of CCT for children with learning differences. A pioneering study conducted by Klingberg et al. [23] examined whether CCT improved ADHD symptomology in children aged 7–12 years. Participants completed 40 min of adaptive cognitive training for at least 25 days. The Digit Span, span-board task, and Stroop test were administered as measures of verbal WM, visual WM, and inhibition, respectively. Across the board, all measures of cognitive skills improved at the posttest. Critically, parent-reported ADHD symptoms decreased at the posttest [23]. These findings were among the first to show that CCT was effective in building specific cognitive abilities in children with a neurodevelopmental disorder.

Since this early study, a plethora of work has documented similar effects as a result of CCT. Among these, for example, is a study conducted by Johann and Karbach [24] in which improvements in cognitive skills (e.g., WM, inhibition, CF) were examined after children engaged with a game-based CCT program. School-age children (ages 8–11 years) participated in 10 h of CCT over a 5-week period. Following training, there were significant improvements in measures of WM, inhibition, and CF—all of which tend to be hindered in children with learning differences [24]. Cumulatively, these studies show support for the effectiveness of CCT in improving skills in children with learning differences. For an extended review, see [25].

### 1.4. Extending CCT Findings

Although there is evidence of the efficacy of CCT in remediating cognitive ability deficits, this body of work is not without criticism [25]. Two significant criticisms center around (a) the strength and consistency of cognitive skill improvement and (b) the contexts in which training takes place. Specifically, while cognitive improvements are demonstrated following CCT, many have questioned whether the improvements are robust enough to show long-term effects. Further, much of the existing CCT work is completed in a variety of physical settings (e.g., the home or a clinical office) and under less-controlled training conditions. This calls into question the veracity of the findings. Relatedly, there is the question of how well the training may transfer to the setting where it is supposed to have an impact (i.e., the school) when it is completed somewhere other than a school setting. Thus, there is considerable value in addressing these criticisms through CCT programs that (a) have an extended duration and, therefore, might strengthen positive results and (b) are implemented in a school environment so that intervention can occur where students are present daily and need to use the cognitive abilities that are being developed.

Work addressing the contextual nature of CCT is under way in the literature. One set of studies [26,27] has implemented school-based CCT interventions for children with learning differences. In one study, a group of students received WM training during the school day while the control group participated in an out-of-class activity not designed to improve cognitive skills. Results from measures of verbal and visual WM administered before and after the training program showed that both verbal and visual WM improved at the posttest for the experimental group, but students in the control group did not demonstrate significant change [27]. Further, Wiest et al. [26] found verbal and spatial encoding also improved after CCT, with effect sizes ranging from small to moderate. Together, these findings show that CCT is a feasible intervention for remediating critical cognitive skills in the school setting among children with learning differences [26]. Similar findings are reported elsewhere [28,29].

While the studies cited above provide some emerging evidence of training efficacy in the school setting, the issue regarding the strength and consistency of CCT effects still needs to be addressed within the literature. That is, more research is clearly needed in schools to demonstrate strong and consistent training effects on neuropsychological and achievement variables. Moreover, additional studies are needed to document the effectiveness of more complex training paradigms (e.g., training that involves more than one computer-based activity).

### 1.5. The Current Study

To corroborate and extend the studies above, the current study will test the efficacy of implementing a school-based CCT intervention in children with learning differences, using multiple gamified computer-based activities over an extended period of the school year. Specifically, the goal is to determine whether CCT interventions that implement these characteristics (i.e., within schools and longer training periods) significantly improve WM, CF, and PS. Moreover, we are exploring the implementation of two types of training programs designed to target different cognitive skills. We hypothesize that both CCT training programs will improve children’s WM, CF, and PS at the posttest. Second, we hypothesize that longer training durations will lead to larger effects on cognitive skills. In addressing these research questions, our study will add to the current literature speaking to the viability of implementing CCT interventions in the classroom and examining their effectiveness for enhancing WM, CF, and PS in children.

## 2. Method

### 2.1. Participants

Ninety-five third- to eighth-graders attending a school for children with learning differences participated; this school was chosen as this project focused on the efficacy of CCT among students with learning challenges. Among the students, 65 were male and 30 were female; the average age was 11.66 years (SD = 1.69). Most of the children had previously received a psychoeducational and/or neuropsychological evaluation with a resulting diagnosis (or diagnoses). Typical diagnoses included the following: reading disorder, writing disorder, attention deficit hyperactivity disorder, and/or some type of emotional disturbance (e.g., anxiety disorder). All students had normal visual acuity or corrected to normal acuity. Participants were predominantly Caucasian, and their families were of moderately high to high socioeconomic status. Each of the participant’s parents/guardians had chosen this school site (as opposed to their local public school) for the child’s educational experience. All students were treated in accordance with the APA Ethics Code of Conduct.

### 2.2. Materials

Participants’ neuropsychological capabilities were evaluated with subtests from two norm-referenced clinical measures: the Wechsler Intelligence Scale for Children-V (WISC-V) and the Delis–Kaplan Executive Function System (DKEFS).

Wechsler Intelligence Scale for Children-V. The WISC-V is a measure of cognitive abilities. A child’s cognitive abilities are conceptualized as the combination of verbal comprehension, visual-spatial abilities, fluid reasoning, working memory, and processing speed. Subtests from the working memory (WM) and processing speed (PS) indices were utilized in this project; specifically, the Digit Span task (WM), Coding (PS), and Symbol Search (PS) subtests were administered. Indirectly, each of these subtests provides an indicator of short-term attention. The Digit Span task asks a child to recite verbally presented strings of numbers in forward order (i.e., in the same order as numbers were presented), backward order (i.e., in reverse order from how numbers were presented), and in sequenced order (i.e., in counting order). Coding is a timed task that requires the child to associate a shape with a picture or a number; then, as quickly as possible, they draw the shape that is associated with a picture (for younger children) or a number (for older children). Children have two minutes to complete as many items as they can. Symbol Search requires an individual to determine if a target shape (for younger children) or if one of two target shapes (for older children) is present in a set of shapes shown adjacent to the target. This is a timed task; thus, children complete as many items as possible within a two-minute period. Performance on the WISC-V subtests is described in the form of scaled scores with a mean of 10 and a standard deviation of 3.

Delis–Kaplan Executive Function System. The DKEFS is a norm-referenced measure of verbal and nonverbal executive function. The Trail Making task (a measure of cognitive flexibility or switching) was utilized in this project. Trail Making is a 5-part task composed of visual scanning, number sequencing, letter sequencing, switching, and motor speed administered in the order noted here. The switching task is used as the indicator of cognitive switching. In the switching task, a child connects circles using an alternating sequence of numbers and letters as quickly as possible—that is, the circles are connected in a 1-A-2-B-3-C… pattern. Performance on the Trail Making task is described via a scaled score with an average of 10 and a standard deviation of 3. Each of the five trials of the Trail Making task is an indirect indicator of short-term attention.

Computerized Cognitive Training (CCT). Two CCT programs developed by the University of California at Riverside Brain Game Center were employed in this project. Recollect the Study and Sightseeing are iPad-based adaptive activities that provide cognitive training activities in a gamified format. Recollect includes a n-back task along with a memory span activity. In the n-back task, the player “travels” through space collecting gems using a specified rule (e.g., collect a gem if it matches a gem one back or collect a gem if it matches a gem two back, etc.). On the memory span task, the individual is asked to recall in order (on the iPad screen) a series of shapes that were just presented. Sightseeing is a visual processing game in which the participant identifies targets on an opaque background as fast as possible. During the activity, distractors are included as the child searches for targets as fast as possible. Both Recollect and Sightseeing are adaptive in that the level of challenge is adjusted automatically for each player in real time; thus, children are always experiencing an activity that is tied to their current performance.

### 2.3. Procedure

All parents (or guardians) of students attending the school were provided with a flyer describing the project along with a consent form. Following receipt of consent to participate, each student was pretested on the Digit Span, Coding, Symbol Search, and Trail Making tasks to establish a baseline for neuropsychological abilities. Following pretesting, participants were randomly selected to participate in either Recollect or Sightseeing. The CCT program was designed as a 20 min daily experience Monday through Thursday until 6 hours of training was attained; Fridays were used as a make-up day for students who missed a Monday–Thursday session. All participants received 12 h of cognitive training in counterbalanced order across the two games. The CCT occurred during the school day in each participant’s respective classroom. Teachers in each classroom administered the CCT, monitored student progress, and offered emotional support as needed. During the first training session, teachers provided students with directions on how to play the game that was assigned. Thereafter, a teacher provided assistance to participants as needed. Following six hours of CCT, each student was readministered the neuropsychological measures (posttest 1). Subsequently, students began training on the game that they had not previously trained on. The training regimen was identical; that is, teachers implemented 20 min sessions each day until 6 hours of training was attained. The neuropsychological indices were administered again following the second 6 h training window (posttest 2).

Teacher Training. Because the teachers were instrumental to training efficacy, they each received training on the CCT games from research personnel before the 12 h program (for participants) was initiated. Three hours and forty minutes of school-based, interactive preparation was provided to each teacher. This experience included a 90 min introduction to the games followed by the teachers playing 10 min of each game while being observed by research personnel. Feedback was provided to teachers as they practiced the games. Any questions were answered during the preparation session. Thereafter, teachers were assigned an iPad and asked to play another 2 h and 20 min on their own over the course of a week; thus, there was 1 h and 10 min of practice for each game. After independent practice, teachers reconvened with research personnel; any questions from the teachers were addressed before the actual CCT began with the participants.

### 2.4. Design/Statistical Analysis

A pretest-posttest experimental design was implemented. Participants completed two training games (Recollect the Study and Sightseeing). The order in which participants completed the games was counterbalanced. Condition 1 represents participants who completed Recollect the Study first and then Sightseeing. Condition 2 represents participants who completed Sightseeing first and then Recollect the Study. Measures of WM (Digit Span), CF (Trail Making task), and PS (Coding and Symbol Search) were given at the pretest, posttest one (after 6 h of training), and posttest two (after 12 h of training).

One-way repeated measures ANOVAs were conducted for each of the measured variables for each condition. For each condition, differences among the pretest, posttest one, and posttest two measures were analyzed. Scores on Digit Span, Trail Making, Coding, and Symbol Search were entered as the dependent variables. Cohen’s d was also calculated to indicate effect size.

## 3. Results

Results for 95 participants are reported here. First, results for Condition 1 (*n* = 50) will be presented. Subsequently, results for Condition 2 *(n* = 45) will be presented. As stated earlier, one-way repeated measures ANOVAs were conducted for all analyses. Effect sizes are represented by Cohen’s d where a small effect is 0 to 0.49, a medium effect is 0.50 to 0.79, and a large effect is above 0.80. See Table 1 below for descriptive statistics.

### 3.1. Condition 1

#### 3.1.1. Working Memory

**Figure 1:**
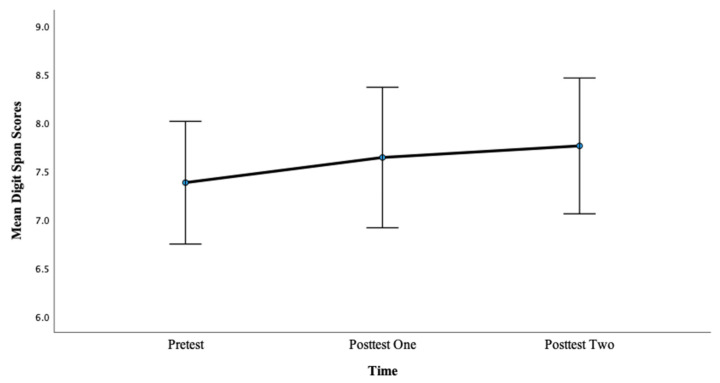
Mean digit span scores at the pretest, posttest one, and posttest two for Condition 1.

For Condition 1, there was no significant main effect of time on Digit Span scores, *F*(2, 98) = 0.90, *p* = 0.410, η^2^ = 0.018. As such, the pairwise comparisons were not significant. In sum, there were no differences between the pretest, posttest one, and posttest two measures of WM for Condition 1. See Figure 1 below.

#### 3.1.2. Cognitive Flexibility

**Figure 2:**
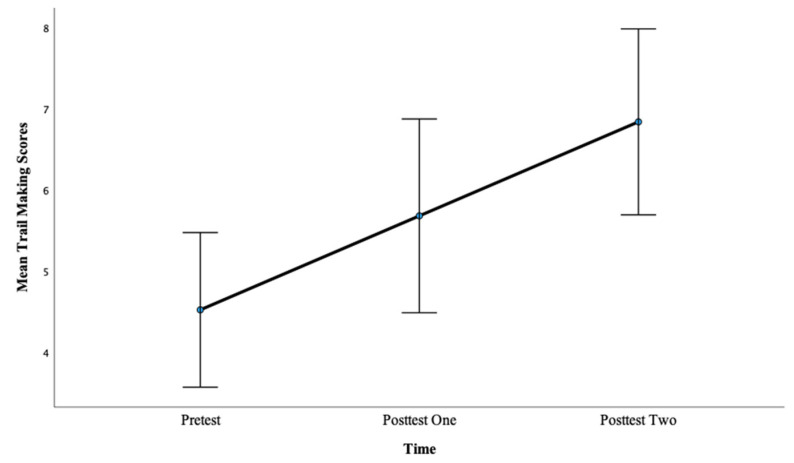
Mean Trail Making scores across the pretest, posttest one, and posttest two for Condition 1.

For the Trial Making task, there was a significant main effect of time, *F*(2, 98) = 8.48, *p* < 0.001, η^2^ = 0.15. Pairwise comparisons showed that there was a significant difference between pretest mean scores (M = 4.52) and posttest one mean scores (M = 5.68), *p* < 0.05, with a large effect, d = 2.10. Also, there was a significant difference between pretest mean scores (M = 4.52) and posttest two mean scores (M = 6.84), *p* < 0.001, with a large effect, d = 4.30. This finding suggests that more hours of training lead to greater gains on CF as evidenced by a larger effect size after 12 h of training. See Figure 2 below.

#### 3.1.3. Processing Speed

**Figure 3:**
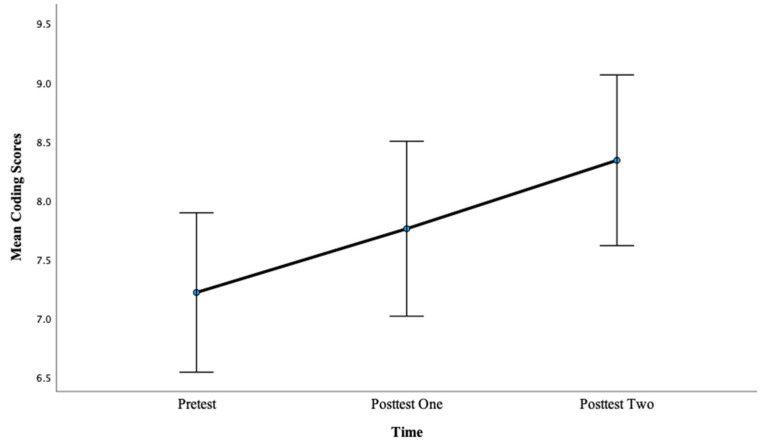
Mean coding scores across pretest, posttest one, and posttest two for Condition 1.

For Coding scores, there was a significant main effect of time, *F*(2, 98) = 9.30, *p* <0.001, η^2^ = 0.16. Pairwise comparisons showed that there was a significant difference between pretest mean scores (M = 7.22) and posttest one mean scores (M = 7.76), *p <* 0.05, with a large effect, d= 2.13. Also, there was a significant difference between pretest mean scores (M = 7.22) and posttest two mean scores (M = 8.34), *p* < 0.001, with a large effect, d = 4.01. This finding suggests that more hours of training lead to greater gains in PS scores as evidenced by a larger effect size after 12 h of training compared with 6 h of training. See Figure 3 below.

**Figure 4:**
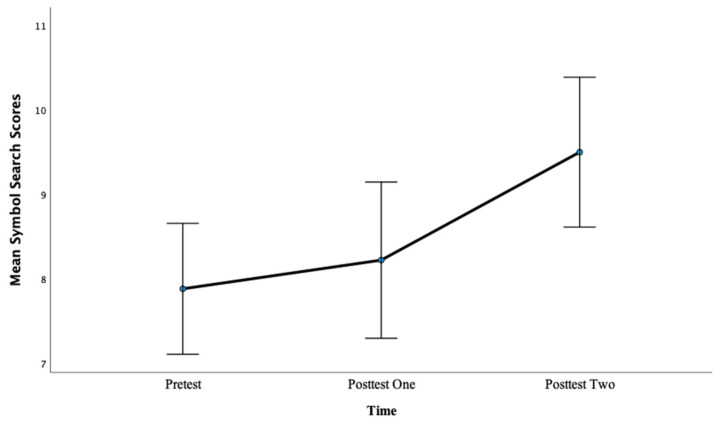
Mean symbol search scores across the pretest, posttest one, and posttest two for Condition 1.

Similarly, for Symbol Search scores, there was a significant main effect of time, *F*(2, 98) = 9.38, *p* < 0.001, η^2^ = 0.16. Pairwise comparisons showed that there was not a significant difference between pretest mean scores (M = 7.88) and posttest one mean scores (M = 8.22), *p =* 0.41, with a large effect, d = 0.83. However, there was a significant difference between pretest mean scores (M = 7.88) and posttest two mean scores (M = 9.50), *p* < 0.001, with a large effect, d = 4.09. This finding suggests that more hours of training lead to greater gains in PS scores as evidenced by a larger effect size after 12 h of training compared with 6 h of training. In addition, the pre- to posttest two difference was the only significant difference regarding time for Symbol Search in this condition, suggesting that for this specific measure, more hours of training were required to produce an effect. See Figure 4 below.

### 3.2. Condition 2

#### 3.2.1. Working Memory

**Figure 5:**
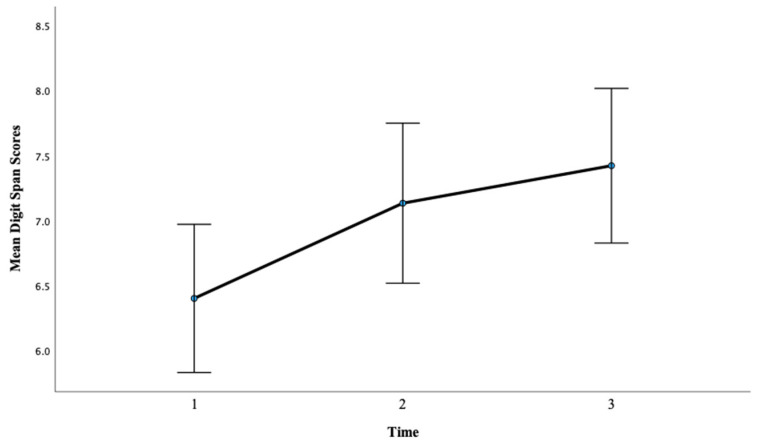
Mean digit span scores for Condition 2 at the pretest, posttest one, and posttest two.

There was a significant main effect of time on Digit Span scores, *F*(2, 88) = 6.31, *p* < 0.01, η^2^ = 0.13. Pairwise comparisons showed that there was not a significant difference between pretest mean scores (M = 6.40) and posttest one mean scores (M = 7.13), *p* = 0.06, with a large effect, d = 2.41. However, there was a significant difference between pretest mean scores (M = 6.40) and posttest two mean scores (M = 7.42), *p* < 0.01, with a large effect, d = 3.39. This finding suggests that more hours of training lead to greater gains on WM scores as evidenced by a larger effect size after 12 h of training compared with 6 h of training for this condition. In addition, the pre- to posttest two difference was the only significant difference regarding time for Digit Span scores in this condition, suggesting that for this specific measure, more hours of training were required to produce an effect. See Figure 5 below.

#### 3.2.2. Cognitive Flexibility

**Figure 6:**
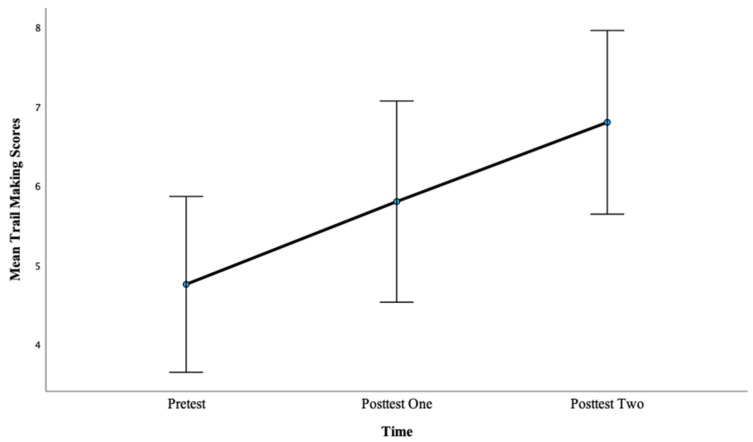
Mean Trail Making scores for Condition 2 at the pretest, posttest one, and posttest two.

There was a significant main effect of time on the Trail Making task scores, *F*(2, 88) = 7.84, *p* < 0.001, η^2^ = 0.15. Pairwise comparisons showed that there was not a significant difference between pretest mean scores (M = 4.76) and posttest one mean scores (M = 5.80), *p* = 0.13, with a large effect, d = 2.06. However, there was a significant difference between pretest mean scores (M = 4.76) and posttest two mean scores (M = 6.80), *p* < 0.01, with a large effect, d = 4.44. This finding suggests that more hours of training lead to greater gains in CF scores as evidenced by a larger effect size after 12 h of training compared with 6 h of training for this condition. It is also the case here that more hours of training were necessary to produce a pre- to posttest improvement. See Figure 6 below.

#### 3.2.3. Processing Speed

**Figure 7:**
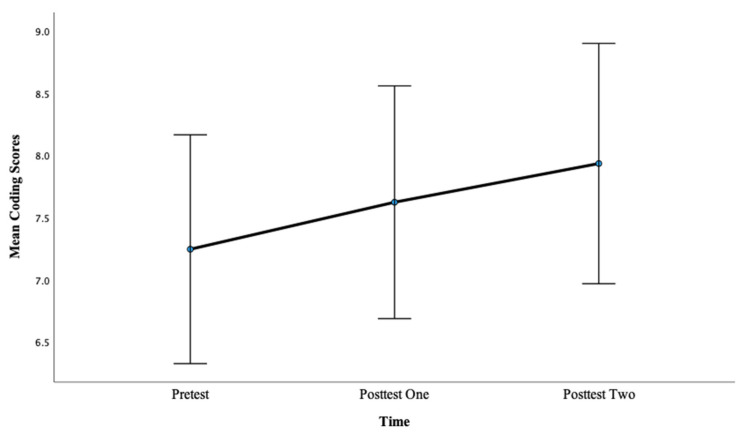
Mean Coding scores for Condition 2 at the pretest, posttest one, and posttest two.

There was a significant main effect of time on Coding scores, *F*(2, 88) = 3.57, *p* < 0.05, η^2^ = 0.13. Pairwise comparisons showed that there was not a significant difference between pretest mean scores (M = 7.24) and posttest one mean scores (M = 7.62), *p* = 0.34, with a large effect, d = 1.61. However, there was a significant difference between pretest mean scores (M = 7.24) and posttest two mean scores (M = 7.93), *p* < 0.01, with a large effect, d = 2.51. This finding suggests that more hours of training lead to greater gains in PS scores as evidenced by a larger effect size after 12 h of training compared with 6 h of training for this condition. Again, a significant improvement from the pretest to posttest was only obtained after 12 h of training for Coding in this condition, suggesting that longer training engagement is important. See Figure 7 below.

**Figure 8:**
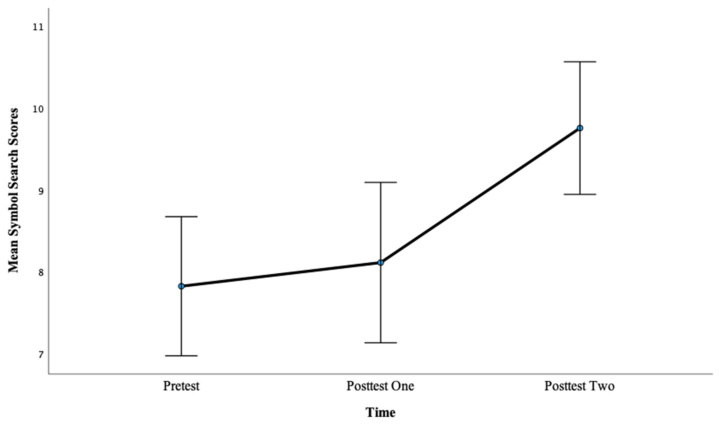
Mean Symbol Search scores for Condition 2 at the pretest, posttest one, and posttest two.

Additionally, there was a significant main effect of time on Symbol Search scores, *F*(2, 88) = 15.75, *p* < 0.001, η^2^ = 0.26. Pairwise comparisons showed that there was not a significant difference between pretest mean scores (M = 7.82) and posttest one mean scores (M = 8.11), *p* = 1.00, with a medium effect, d = 0.72. However, there was a significant difference between pretest mean scores (M = 7.82) and posttest two mean scores (M = 9.76), *p* < 0.001, with a large effect, d = 5.87. This finding also suggests that more hours of training lead to greater gains in PS scores as evidenced by a larger effect size after 12 h of training compared with 6 h of training for this condition. In addition, the pre- to posttest two difference was the only significant difference regarding time for Symbol Search in this condition, suggesting that for this measure, more hours of training were required to produce an effect. See Figure 8 below.

## 4. Discussion

The current study sought to extend the literature on CCT by examining the efficacy of a school-day intervention program in children with learning differences. Specifically, one goal was to investigate the effectiveness of CCT in improving neuropsychological abilities such as working memory, cognitive flexibility, and processing speed. The second objective was to provide evidence that extended training with gamified cognitive activities is important with regard to the outcome measures. This study utilized two different computer-based activities for an extended period of the school year with all training occurring during the school day in each participant’s respective classrooms. The findings of the current investigation indicate that CCT is efficacious in building core cognitive abilities that are critical to school success. Moreover, there is evidence to indicate that more CCT training leads to greater treatment effects.

### 4.1. Effectiveness of CCT in Improving WM, CF, and PS

The current results provide strong evidence of the CCT effectiveness in enhancing WM, CF, and PS at two different levels. First, with regard to statistically significant conclusions, seven of the eight ANOVAs indicated that each of the cognitive abilities improved by the conclusion of the two 6 h training periods; the only nonsignificant finding was present for WM among participants in Condition 1 (Recollect training, then Sightseeing). These results are consistent with previous research that documents positive treatment effects of CCT [26,27]. As such, this study provides additional confirmation that CCT is a viable means of remediating neuropsychological weaknesses. Importantly, the methodological approach illustrated in this project indicates that CCT can be implemented in the setting (i.e., the classroom) in which the trained skills are utilized; thus, there is notably less concern with the effective transfer of training as noted in previous research (see [25]). Furthermore, the results of this and other studies that engage students in CCT within the school-day curriculum (see [26,27]) highlight how the higher value of CCT may be efficiently delivered to produce the greatest influence.

Beyond the nomothetic results, the effect size indices reported in this project further highlight the practical effectiveness of CCT. Across the 16 Cohen’s d reported, 15 were of a magnitude that would be described as a “large effect”. The only effect size estimate that was not a “large effect” was found for the pre- to posttest one change in Symbol Search (processing speed) performance for Condition 2 (Sightseeing training, then Recollect) participants; this Cohen’s d was categorized as “moderate”. Though not an indicator of statistical significance, Cohen’s d metric provides important and valuable real-world information regarding the benefit of this type of intervention.

### 4.2. Is Extended CCT Training Important?

The second objective of this project was to examine a critical issue in CCT research that has received inadequate attention. In particular, this study has addressed whether extended training leads to greater treatment effects. Our hypothesis has been supported: the results indicate that more training leads to greater improvement in the measured cognitive variables. Specifically, of eight possible pairwise comparisons after six hours of training (i.e., pre- vs. posttest one) for the four outcome variables (Digit Span, Trail Making, Coding, Symbol Search), only two were statistically significant. There was a significant improvement in CF and Coding (PS) among participants in Condition 1 (i.e., Recollect training, then Sightseeing). On the other hand, of eight possible pairwise comparisons at the end of 12 h of training (i.e., pre- vs. posttest two) for the three outcome variables (WM, CF, and PS), seven were statistically significant. In other words, regardless of condition, participants generally exhibited significant gains in working memory, cognitive flexibility, and processing speed after 12 cumulative hours of training. This suggests that the effects of CCT are stronger after extended training, resulting in substantial cognitive changes. Overall, these findings are strong evidence that extended cognitive training is important. These findings are a valuable extension of the CCT literature as there has been little research that has focused on this treatment effect variable.

### 4.3. Implications of the Current Study

There are a number of important implications associated with the current project. First, the results reported here provide further evidence for the plasticity of neuropsychological abilities. As noted earlier, it was a long-standing assumption that cognitive abilities are relatively fixed until cognitive training research demonstrated otherwise (see [20,21]). The current results provide further evidence that working memory, cognitive flexibility, and processing speed are, in fact, malleable; thus, they can be “exercised” in a manner that results in more well-developed skills. Of course, stronger neuropsychological skills have important positive consequences with regard to students’ academic performance (see [6,8]). Second, this project illustrates the efficacy of CCT among school-age children with learning differences—that is, cognitive training works. An especially unique contribution of this work is that we demonstrate that CCT can be integrated seamlessly into a school day. In many ways, this may increase the effectiveness of training as it occurs in the same setting in which the cognitive skills (being trained) are tapped on a daily basis. It is important to have more CCT research conducted in the school setting (during the school day) to fully understand its promise to enhance abilities that are central to strong school-related outcomes. Last, the current findings highlight important considerations regarding ongoing training. We demonstrated that significant improvements occurred in the measured cognitive abilities after 12 total hours of training; however, the same was not generally true after 6 hours of training. This suggests that treatment effects for CCT are enhanced with consistent, extended training during students’ school days. Future research will need to examine this little-addressed issue in a more prominent manner.

With regard to the broader issue of CCT efficacy for students in general, it will be important to address the effects of CCT in children who are not identified with learning differences. That is, having a sample of “typically-” and “atypically”-developing participants will facilitate a finer level of analysis of CCT effects.

## 5. Conclusions

This project is a unique combination of neuropsychological and cognitive research with a particular focus on CCT. The findings show that cognitive abilities are changeable, that CCT is effective in building cognitive abilities, and that intervention effects can be enhanced with sustained, consistent training implemented within a school day. Future work should focus on corroborating the current results as there are critical benefits of that knowledge not only for researchers but also for practitioners.

## Figures and Tables

**Table 1:** Descriptive statistics for all conditions and cognitive outcome measures across time.

Condition	Time	Digit Span	Trail Making	Coding	Symbol Search
Condition 1					
	Pretest	7.38 (2.23)	4.52 (3.36)	7.22 (2.38)	7.88 (2.73)
	Posttest one	7.64 (2.55)	5.68 (4.21)	7.76 (2.61)	8.22 (3.26)
	Posttest two	7.76 (2.47)	6.84 (4.04)	8.34 (2.54)	9.50 (3.12)
Condition 2					
	Pretest	6.40 (1.90)	4.76 (3.69)	7.24 (3.06)	7.82 (2.83)
	Posttest one	7.13 (2.05)	5.80 (4.20)	7.62 (3.11)	8.11 (3.26)
	Posttest two	7.42 (1.98)	6.80 (3.86)	7.93 (3.21)	9.76 (2.70)

Note. *n* = 95. Standard deviations are shown in parentheses.

## Data Availability

The data presented in this study are available on request from the corresponding author.
